# Tailoring Biologic or Immunomodulator Treatment Withdrawal in Inflammatory Bowel Disease

**DOI:** 10.3389/fmed.2019.00302

**Published:** 2020-01-08

**Authors:** Edouard Louis

**Affiliations:** Department of Gastroenterology, University and CHU Liège, Liège, Belgium

**Keywords:** Crohn's disease, ulcerative colitis, treatment withdrawal, prediction, relapse

## Abstract

There is currently no cure for inflammatory bowel disease. Most recent treatments and treatment strategies allow for healing intestinal lesions and maintaining steroid-free remission in a subset of patients. These patients and their doctors often ask themselves whether the treatment could be withdrawn. Several studies in both Crohn's disease and ulcerative colitis have demonstrated a risk of relapse, which varies between 20 and 50% at 1 year and between 50 and 80% beyond 5 years. These numbers clearly highlight that stopping therapy should not be a systematically proposed strategy in those remitting patients. Nevertheless, they also indicate that a minority of patients may not relapse over mid-term and that those who have relapsed may have benefited from a drug-free period before being treated again for a new cycle of treatment. In this context, it would be good to optimally select patients who can be candidates for a successful treatment withdrawal. The criteria impacting this decision are as follows: the risk of relapse (linked to factors like mucosal healing and biomarkers), the consequence of a potential relapse, the tolerance and potential side effects of therapy, patients' priorities and preferences, and the costs. Integration of these parameters allows for the proposal of a decisional algorithm that may help the patients and doctors to make an appropriate decision for their individual case.

## Introduction

The cure for a disease is logically considered as a main situation where a treatment withdrawal can be decided. However, there is currently no cure for inflammatory bowel disease (IBD). In our current conception, those are multifactorial polygenic diseases (1). Therefore, a cure is highly unlikely. What we could imagine is to be able to sufficiently modify the environment to be able to stop the ongoing immuno-inflammatory process (2). There are two limitations to this possibility: first, the self-perpetuation of inflammation would be installed and not be possible to stop even retrieving environmental triggers, and second, the cumulated tissue damage would generate symptoms. This second point should not be an obstacle to treatment withdrawal but would rather require complementary symptomatic treatments. Beyond this, a treatment withdrawal would also make sense when the benefit of the treatment is lower than its risk and/or cost. Most often, it is considered that cost here is a political health care system or a pragmatic insurance company decision that cannot be made at the level of individual patients. The situation where it could be decided on an individual patient basis is when the patient is not covered for his/her medical fees and has to decide himself or herself how to spend money, including for health care. This situation is very rare in western Europe. Nevertheless, public or private health institutions have important decision to make in this field. For them, the benefit/cost ratio is certainly relevant and has to be taken into account. For the physician, it is thus usually the benefit/risk ratio that is dominant. Assessing this is not an easy task as the physician thus needs to integrate and compute at the same time the risk of ongoing drug therapy and the benefit of this therapy. Furthermore, the risk linked to treatment withdrawal is not limited to the risk of relapse. We also have to consider the probability of rapidly recovering remission after retreatment, and if the response to retreatment was not appropriate, the consequences of the disease flare, including the risk of surgical resection. It is even more complicated as the physician should also integrate the patient's preferences and priorities. Indeed, the acceptance of the risk of side effects and the risk of disease progression may vary from patient to patient.

The aims of this review article are to illustrate the most important factors to consider when contemplating treatment withdrawal in IBD and to propose a way to integrate these various factors. The benefit/risk and benefit/cost ratios of mesalazine has been recently reviewed and probably remains positive over time (3). Therefore, we will focus on biologic and immunomodulator withdrawal. As far as biologic therapy is concerned, there are currently essential data on anti-tumor necrosis factor (anti-TNF) and concerning an immunomodulator, essentially purines.

## The Risk of Relapse After Treatment Withdrawal in IBD

The risk of relapse after treatment withdrawal is probably a point that has been best documented. Overall, both in Crohn's disease (CD) and in ulcerative colitis (UC), the risk of relapse after stopping anti-TNF is around 50% over 1–2 years (4, 5). It is probably increasing with time of follow-up and has been described around 70–80% in CD after 7–8 years (6). Withdrawal of immunomodulator seems to be associated with a slower relapse risk (7). It has been estimated to be around 20–30% after 1–2 years. However, here again it progresses with time and reaches >50% after 5 years (8). After retreatment, over the short term, most of the patients respond to resuming both anti-TNF or immunomodulator (4, 5, 9). For anti-TNF, a small proportion will lose response over time, but a substantial number of them are still effectively treated with the same drug more than 5 years later (6). In UC, up to 10% of withdrawn patients may have to undergo colectomy within 1 year after anti-TNF withdrawal (10), while this proportion seems lower in CD with also 10–15% but only over 7–8 years (6). These risks are too high to propose a treatment withdrawal in all patients reaching sustained steroid-free remission in IBD. This assertion is reinforced by patients' survey highlighting the fact that among them, the majority would only accept a maximum risk of relapse of 25% (11). According to this, we should try to identify a subpopulation with a risk of relapse lower than 25%. Predictors of relapse have been studied in many studies with anti-TNF and immunomodulators. No data are available for vedolizumab or ustekinumab. These predictors have recently been extensively reviewed, and results are heterogeneous (3–5, 12). This heterogeneity is explained by the heterogeneity of the study populations, including the differences between prospective trials and retrospective analyses of routine practice populations. In routine practice, the selection of the population for treatment withdrawal is more stringent, focusing on, for example, patients in endoscopic remission, while prospective trials may also have included patients still having endoscopic lesions. A certain heterogeneity can also be explained by predictors that have been studied, particularly in retrospective studies where only a limited amount of variables was available. Results were also different when considering the withdrawal of anti-TNF or immunomodulator and in CD or in UC. In general, predictors have been more difficult to disclose in UC than in CD. In the largest retrospective study so far, while a series of predictors could be found for CD, none was found for UC (12). Among the most prominent predictors are the direct or indirect signs of persisting disease activity: endoscopic lesions, elevated blood markers of inflammation (C-reactive protein), and elevated stool markers of inflammation (fecal calprotectin) (3–5). Other prominent predictors are linked to ongoing treatment: co-treatment with an immunomodulator and low or undetectable trough level of anti-TNF were associated with a lower risk of relapse when stopping this anti-TNF (3–5). According to this, persisting endoscopic lesions and trough level of the drug are often considered as key factors for clinicians to be assessed in clinical practice before considering drug withdrawal. Albeit important, they only represent part of the problem. Indeed, in the STORI cohort, even in patients with full endoscopic healing, the relapse rate after infliximab withdrawal was 30% over 1 year (as compared to 45% in the general population and 10% in the low-risk group). Likewise, a low or undetectable trough level of infliximab has been associated with a decreased risk of relapse upon withdrawal. This makes sense and probably corresponds to situations where infliximab has a minor impact on the maintenance of remission. It is, however, not so straightforward, as a low trough does not necessarily mean no effect of the drug. This drug may still generate relevant exposition linked to peak concentration and area under the curve of this concentration over 4–8 weeks. In the STORI cohort, the infliximab level was not associated with the risk of relapse in univariate analysis but was only selected in the multivariate model. Other factors have been proposed, but they either also indirectly reflect ongoing disease activity or current treatment or are more difficult to explain and need to be confirmed. Smoking, which has often been associated with bad outcome in CD, has only been found predictive of relapse after stopping anti-TNF in one study (3). Histologic remission, which is becoming an important outcome in UC and which is questioned in CD, has not been adequately studied as a predictor after treatment withdrawal in IBD. According to these results, the best candidates for anti-TNF withdrawal would be patients with clinically, biologically, and endoscopically inactive disease and with immunomodulator co-treatment and/or low-undetectable biologic drug level (Table 1). In the STORI cohort, it represented 15–20% of the patients recruited in the trial (9). This gives an estimation of the proportion of patients among those having longstanding steroid-free remission under combination therapy, with a low risk of relapse. However, as the retreatment upon relapse seems safe and effective and as a substantial number of patients may benefit from at least temporary drug withdrawal, the candidates for temporary withdrawal may be more numerous.

**Table 1:** Most important factors favoring treatment withdrawal in IBD.

**Factors associated with a lower risk of relapse**
Mucosal healing (mainly CD and anti-TNF)
Normal CRP (mainly CD)
Low fecal calprotectin (<250 μg/g) (mainly CD and anti-TNF)
Low or undetectable trough levels of biologic treatment (mainly CD and anti-TNF)
Immunomodulator co-treatment (mainly CD and anti-TNF)
**Factors associated with low cumulative intestinal tissue damage**
No complex perianal disease
No severe rectal disease
No intestinal or colonic stricture
No history of intra-abdominal abscess or fistula
Limited extent of the disease in the past
**Factors associated with increased risk of treatment side effects**
Older age (>65 years old)
Co-morbidities favoring infection or the risk of cancer
Side effects attributed to the treatment
**Patient's preference**
Pregnancy
High fear of treatment side effects
Low fear of surgery
Acceptance of relapse risk
**Cost**
Expensive medication
No/insufficient reimbursement

*For the factors associated with a lower risk of relapse, the situations for which evidence is the strongest are put under brackets*.

*IBD, inflammatory bowel disease; CD, Crohn's disease; TNF, tumor necrosis factor; CRP, C-reactive protein*.

## The Consequences of the Relapse After Treatment Withdrawal in IBD

Relapsing after biologic or immunomodulator treatment withdrawal would be a minor problem if a remission could rapidly be re-captured and without disease progression leading to the need of a surgical resection. The situation is obviously much different if the relapse is associated with the development of a complication, like a stricture, an abscess, or a fistula in CD, and an acute severe colitis in UC. For UC, the occurrence of such acute severe colitis remains unpredictable and does not help to tailor the decision (13). In some series, however, the colectomy rate was up to 20% of relapsing patients and is thus an important limitation for this strategy (10). CD patients already having a history of perianal fistulizing disease or intestinal strictures or fistula and abdominal abscess are at risk of recurrence (12, 14). Likewise, patients already operated on have a significant amount of intestinal tissue damage, and the clinician should be very careful not to increase it, particularly when there is a risk of short bowel or a risk of subtotal colectomy or stoma (15). In the published studies, the risk of relapse was particularly high in patients with previous fistulizing perianal disease (14). Probably explaining this, previous studies have illustrated that patients experiencing full clinical closure of their perianal fistulas under anti-TNF treatment usually keep signs of active inflammation in their fistulous tracks and that the full healing and disappearance of these fistulous tracks are very rare (16, 17). Likewise, previous studies have shown a possible increased risk of relapse in patients with a history of intestinal strictures or fistulas (12). In those studies, the risk of developing new strictures, fistulas, or abscess after anti-TNF of immunomudulator withdrawal was not clearly indicated, but in the long-term follow-up of the STORI cohort, with a median follow-up of 7 years, only 18% of the patients developed major complications including the need for surgical intestinal resection and new complex perianal fistulas. According to this, the best candidates for treatment withdrawal would be patients with no history of complex perianal disease; no significant and recent stricture, fistula, or intra-abdominal abscess; and no extensive surgical resection in CD (Table 1). Likewise, patients with left-sided UC or proctitis could be better candidates than those with pancolitis. Age is also important to take into account as young patients will have to live longer with their disease and are thus at increased risk of complications and cumulative intestinal tissue damage.

A key element in case of relapse after treatment withdrawal is the ability to re-capture the remission with the same drug. This may be jeopardized by drug immunogenicity for biologics and the development of anti-drug antibodies. These anti-drug antibodies have been associated with transient drug withdrawal, particularly with infliximab. This was particularly pronounced in early experience with infliximab when only induction treatment was given, followed by on-demand therapy. Scheduled treatment and immunomodulator co-treatment have clearly decreased immunogenicity, and in the STORI trial, only a few patients developed anti-drug antibodies and none experienced acute severe infusion reaction when resuming therapy (9). However, in the STORI trial, due to this theoretical risk of allergic reaction when restarting infliximab, a steroid infusion was given before resuming infliximab and the first infusions were performed at a slower pace, with a small amount of the drug infused during the first hour. This is still our practice today, although no controlled clinical trial validated this strategy. The risk of immunogenicity with more recent biologics in the context of transient drug withdrawal is less well-documented.

## The Risk of Ongoing Treatment in IBD

The risk and tolerance of ongoing treatment is primarily influenced by age and comorbidities (18). The risk of severe infection under anti-TNF therapy has been shown to be significantly higher in patients older than 65 years (19). Likewise, anti-TNF and purine analogs are associated with an increased risk of lymphoma (20). The relative risk has been estimated around 2 for anti-TNF and around 2–4 under purine analogs, while it culminated at 4–6 under combined therapy (20). However, this risk increases with age, leading to a substantial number (albeit still low in absolute numbers) of patients older than 65 years developing lymphoma under these drugs (21). Purines have also been associated with other forms of cancers, including skin cancers and urinary tract cancers (22, 23). For these reasons, most clinicians now try to decrease the use of purine analogs beyond 60–65 years of age. The impact of anti-TNF and other biologics on other cancers is not well-documented, apart from skin cancers and perhaps melanoma under anti-TNF (24). Nevertheless, due to the increased risk of cancers in aging people, drugs with a systemic immunosuppressive effect should be used with caution. Some comorbidities may also require attention. It includes chronic obstructive pulmonary disease, which is associated with an increased risk of bronchopulmonary superinfection (25). Again, this may be increased by drugs having a systemic immunosuppressive effect.

Another aspect is mild intolerance to the drug, like some skin manifestations under anti-TNF therapy (26). Most often, these manifestations are not sufficient *per se* to lead to treatment interruption if the benefit of the treatment remains significant (27). However, in some situations, it may represent one argument among many others that may influence the decision.

Therefore, from this point of view, the best candidates for treatment withdrawal would be patients with some degree of intolerance to the drug, or older patients (usually above 60–65 years of age) or those having comorbidities increasing the risk of infection or cancer (Table 1).

## The Cost of Ongoing or Stopping Treatment Strategies

The cost of ongoing treatment will vary very much depending on its nature: biologic therapy, biosimilar, or immunomodulator. Although recent studies have demonstrated that a growing part of the cost of management of IBD was linked to biologic therapy, this did not take into account the spared costs due to a decrease of hospitalizations or surgeries (28). In early studies with infliximab, the drug was considered as cost-effective in CD but only for one or a few years of therapy, the cost-effectiveness being not demonstrated beyond this duration (29). A more recent study specifically looked at the cost-effectiveness of a strategy of cycles of biologic therapies, including periods of withdrawals when the patients were in long-standing remission (30). This study showed that the cost-effectiveness of continuous therapy was favorable at some drug cost thresholds. Interestingly, with biosimilars, these thresholds have recently been reached in several European countries. The situation for biologics paid at the full price is different, and for those, the continuous treatment is generally less cost-effective than cycles of biologic treatment. The price of purine analogs is usually so low that continuous therapy is most often cost-effective.

## Patients' Preferences and Priorities

Due to personal views on the disease and its treatments, patients may be more keen to accept consequences or complications of the treatment or of the disease itself. The choice between medical therapy and surgical therapy (which may be a consequence of withdrawing therapy), for example, may vary among patients. In a dedicated patients' survey, it was shown that the risk of severe infection or lymphoma that the patient would accept to be in remission would vary very much but would be usually higher than the one accepted by their doctors (31). More specifically, concerning treatment withdrawal, it was shown that the patients would usually prefer to stop immunomodulator than biologic treatments and that the main reason for stopping therapy would be the fear of side effects and particularly cancer (11). As far as the risk of relapse that the patients would accept to be able to stop one of their treatment, the majority would accept up to 25% risk of relapse and up to 5% time with active disease to be able to stop one of their treatments (biologic or immunomodulator) (11). These numbers may serve as landmarks when considering treatment withdrawal. However, some patients would not accept any risk of relapse, while others would be ready to accept very high risk to decrease their therapy (11). These questions should be specifically asked to the patients before considering treatment withdrawal.

Pregnancy represents a particular situation in which treatment withdrawal is often contemplated or at least discussed. A pregnant patient is usually very keen to stop therapy even before the start of pregnancy. However, despite a relatively low amount of evidence, most guidelines consider that almost all treatments can be continued during pregnancy, except for methotrexate (32). The consensus is that the worst thing for a pregnancy both for the fetus and the mother is an uncontrolled disease and that everything should be made to keep remission during pregnancy.

From this point of view, the best candidates for treatment withdrawal would be the ones who, after a clear information and understanding of not only the risks linked to treatment withdrawal but also the consequences of continuing therapy, choose to stop this treatment (Table 1).

## Integrative Model to Guide Treatment Withdrawal in IBD

**Figure 1:**
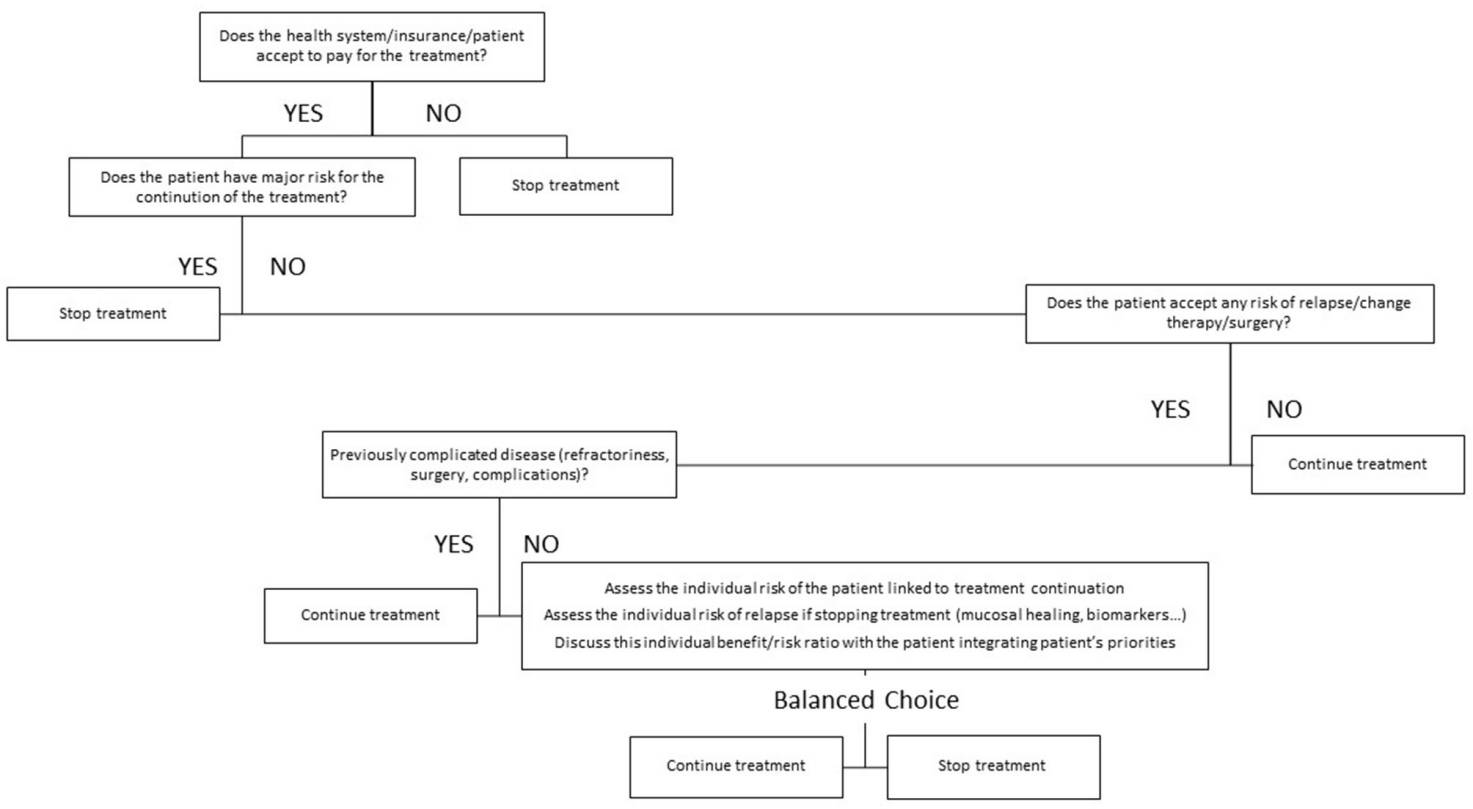
Proposed algorithm for treatment withdrawal decision in inflammatory bowel disease (IBD). This algorithm may provide a hierarchy among the questions and factors that have to be assessed when contemplating treatment withdrawal in IBD. Some factors like the cost and the reimbursement may be specific to some health care systems and jurisdiction.

The decision to withdraw a treatment in IBD is not an easy one and is clearly multi-dimensional. There are several ways to try and integrate these different dimensions. Most sophisticated would include the development of a clinical decision support system (33). Such tools have been developed for other chronic diseases and can integrate several parameters and the positions of several actors involved in the decision process, including the patients. Artificial intelligence can be incorporated in those tools to optimize the decision. They have yet to be developed in the field of IBD. A simple tool could go through a rough and semi-quantitative weighing of the different factors and a graphical representation of the strength of arguments in favor of stopping or continuing therapy (4). This model has been proposed and illustrated in a previous publication dealing with treatment withdrawal in CD. Alternatively, and more simply, typical patients' profiles can be created in whom a decision of either treatment withdrawal or treatment continuation could be the optimal choice (34). Another relatively simple way to proceed would be to create an algorithm incorporating the different dimensions governing the treatment choice. This would require a hierarchy between the different dimensions allowing for building an algorithm driven by successive question. An example of such algorithm is presented in Figure 1.

## Conclusions

Systematic withdrawal of biologic therapy or immunomodulator when the treatment target has been reached is not evidence based and is not advisable. Nevertheless, for some subgroups of patients, it may represent an option associated with optimal benefit-risk and benefit-cost ratio. The decision to withdraw treatment in IBD patients in remission should thus be a tailored approach, mainly taking into account the past clinical history of the patient, the current disease state, the tolerance and risk of side effects as well as patients' preference and priorities. Optimal integration of all these aspects may require specific tools incorporating artificial intelligence. Simpler algorithms may also help the clinician in routine practice.

## Author Contributions

EL conceived, wrote, and prepared the manuscript.

### Conflict of Interest

EL has received fees for: Research Grant: Takeda, Pfizer; Educational Grant: Abbvie, Takeda, Janssen; Speaker Fees: Abbvie, Ferring, MSD, Falk, Takeda, Hospira, Janssen, Pfizer, Celgene; Advisory Board: Abbvie, Ferring, MSD, Takeda, Celgene, Hospira, Janssen; Consultant: Abbvie. The funders had no role in study design, data collection and analysis, decision to publish, or preparation of the manuscript.
